# Unlocking Biochar's Potential: Innovative Strategies for Sustainable Remediation of Heavy Metal Stress in Tobacco Plants

**DOI:** 10.1155/sci5/6302968

**Published:** 2025-01-06

**Authors:** Abdul Ghaffar Shar, Leyi Zhang, Anzhi Lu, Munib Ahmad, Muhammad Saqib, Sadam Hussain, Usman Zulfiqar, Pingping Wang, Lixin Zhang, Mehdi Rahimi

**Affiliations:** ^1^College of Life Sciences, Northwest A&F University, Yangling 712100, Shaanxi, China; ^2^Barani Agricultural Research Station, Fateh Jang, Attock 43350, Punjab, Pakistan; ^3^College of Horticulture, Northwest A&F University, Yangling 712100, China; ^4^Department of Agronomy, Faculty of Agriculture and Environment, The Islamia University of Bahawalpur, Bahawalpur 63100, Pakistan; ^5^Shaanxi Tobacco Scientific Institution, Xi'an 710000, Shaanxi, China; ^6^Department of Biotechnology, Institute of Science and High Technology and Environmental Sciences, Graduate University of Advanced Technology, Kerman, Iran; ^7^Department of Medical Microbiology, College of Science, Knowledge University, Kirkuk Road, Erbil 44001, Iraq

**Keywords:** absorption, biochar, heavy metals, remediation, stress, sustainable, tobacco plant

## Abstract

Tobacco, being a globally cultivated crop, holds significant social and economic importance. Tobacco plants are susceptible to the adverse effects of heavy metals (HMs), particularly cadmium (Cd), which hinders root development, disrupts water balance, and impedes nutrient absorption. Higher concentrations of HMs, especially Cd, naturally accumulate in tobacco leaves due to complex interactions within the plant–soil continuum. The uptake of Cd by plants from the soil is influenced by several factors, including soil type, pH, irrigation water quality, and the chemical composition of the metal involved. Different techniques, such as bioremediation, phytoremediation, and mycoremediation, have been employed to tackle the issue of HMs. The use of biochar offers a practical solution to mitigate this problem. With its large surface area and porous nature, biochar can effectively alleviate HMs contamination. Under biochar application, metal adsorption primarily occurs through physical adsorption, where metal ions are trapped within the pores of the biochar. Additionally, electrostatic attraction, in which negatively charged biochar surfaces attract positively charged metal ions, is another major mechanism of metal remediation facilitated by biochar. In this review, we documented, compiled, and interpreted novel and recent information on HMs stress on tobacco plants and explored biochar's role in alleviating HMs toxicity. By providing a comprehensive review of the persistent threat posed by Cd to tobacco crops and exploring biochar's potential as a remediation measure, this work aims to enhance our understanding of HMs stress in tobacco and contribute to the development of sustainable agricultural practices.

## 1. Introduction

Tobacco (*Nicotiana tabacum* L.) holds significant commercial and agricultural importance globally [1]. As a member of the Solanaceae family, tobacco plants are prone to contamination through the absorption of heavy metals [2]. However, they are particularly vulnerable to the adverse effects of heavy metals, such as Cd, which can inhibit root growth, disrupt water balance, and impede nutrient absorption. The accumulation of high concentrations of heavy metals, particularly Cd, in tobacco leaves is a consequence of intricate interactions between the soil and plant systems [3]. The uptake of Cd by plants from the soil is influenced by numerous factors such as soil type, pH, irrigation water quality, and the chemical composition of the metal itself.

The increasing accumulation of heavy metals in tobacco plants raises severe concerns for both agricultural productivity and human health [4]. Extensive efforts have been made to detect and mitigate the adverse effects of these metals on plants. Traditional techniques have been employed to detect heavy metals, helping to identify potential threats to plant health. However, heavy metals, particularly Cd, tend to accumulate spontaneously and reach elevated concentrations in tobacco leaves. According to Regassa and Chandravanshi [5], tobacco holds significant agricultural importance globally, with notable social and economic value. While Cd is not naturally present in plants or humans, tobacco plants tend to accumulate it due to its toxicity. Smoking cigarettes exposes individuals to Cd [6]. The accumulation of heavy metals in tobacco plants arises from complex interactions between the plants and their soil environment. Tobacco plants absorb metals from the soil to varying degrees, depending on factors such as soil type, pH, irrigation water quality, chemical composition of the metal, and plant variety [7]. The distribution and accumulation of these metals in tobacco leaves reflect the mineral composition of the soil and surrounding environment.

As a result, the metal content of tobacco can vary significantly depending on various factors such as geographical origin, the type of fertilizers used, and specific characteristics like irrigation water quality [8]. Previous studies have examined tobacco leaves to evaluate heavy metal accumulation resulting from the prolonged use of copper fungicides and phosphate fertilizers on farms. Various approaches are utilized to detect heavy metal accumulation in crop plants. Atomic absorption and flame emission spectrophotometry are commonly used to assess levels of Cd, zinc (Zn), manganese (Mn), cobalt (Co), nickel (Ni), and lead (Pb). Similarly, flame emission and atomic absorption spectrophotometry (AAS) are recommended for determining Pb levels [9].

The application of phosphate fertilizer is a widespread practice aimed at boosting agricultural productivity [10, 11]. However, in tobacco crops, the application of phosphorus fertilizer has been shown to increase the levels of heavy metals in the plants [7]. This increase, however, depends on soil type, time, and application rate of this fertilizer [12]. Previous studies have extensively documented the high absorption and transportation of heavy metals such as arsenic (As), mercury (Hg), and Cd in tobacco plants resulting from the use of chemical fertilizers [13].

Soil, a complex mixture of minerals, microorganisms, and organic matter, plays a significant role in the earth's chemical cycles. Studies show that soil contains four times more carbon than plant biomass, contributing to its high-water retention capacity. Moreover, soil offers numerous essential nutrients that support plant growth, development, and yield. However, the composition of soil is constantly changing due to both natural environmental processes and anthropogenic activities [14]. Soil is characterized by its intricate chemical and elemental makeup, comprising minerals, air, water, organic matter, and fossils. It also plays a crucial role in the carbon cycle, as it can store about three times more carbon than the atmosphere and four times more than plant biomass. Numerous studies have demonstrated a strong correlation between soil carbon content and soil fertility, making the quantification of carbon content in field soils in the context of the carbon cycle and global warming [15].

**Figure 1:**
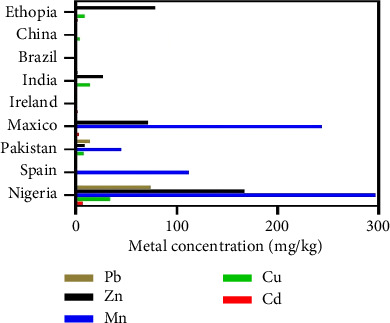
Heavy metals concentrations in tobacco plant collected from various countries [17].

In addition, soil provides essential nutrients, such as N, P, K, Si, Ca, Mg, and S, along with trace elements (e.g., Fe, Cu, Mo, and Ni) for optimal plant functions [14]. However, the uptake of these essential nutrients by plants can be affected by the heavy metal content in soils [16]. In the current era, rapid industrialization has contributed to environmental and soil pollution, with significant amounts of non-degradable artificial materials and metals, including Cd, Cu, and Zn, being released (Figure 1). The uptake of these substances by plants adversely impacts their growth and development. Therefore, assessing the elemental concentrations of heavy metals, minerals, and trace elements in the soil is crucial to supporting sustainable agriculture. Among field crops, tobacco plants are particularly known for absorbing and accumulating high dose of heavy metals, mainly Cd and Hg, in their leaves [13]. Addressing this issue is essential for enhancing the performance of tobacco plants. Various techniques, such as phytoremediation [18] and phytoextraction [19, 20], have been used to tackle this problem [21]. However, some downsides of these techniques have also been reported. On the other hand, the application of biochar, a carbon-rich material derived from pyrolysis, has emerged as a win-win strategy to alleviate heavy metal stress in crop plants. The soil application of biochar can restrain metal(loid)s, reduce their toxic effects, improve soil conditions, and decrease greenhouse gas emissions by altering soil properties [22–24]. Biochar absorbs heavy metals mainly through physical adsorption, ion exchange, electrostatic adsorption, precipitation, complexation, and the combination of these mechanisms [25–27]. However, the efficacy of biochar in heavy metal adsorption depends on its type and the specific metal contaminations in the rhizosphere. For example, Lu et al. [28] studied the mechanism of lead (Pb^2+^) adsorption by biochar produced from sludge and reported that surface precipitation, ion exchange, and surface complexation of functional groups were involved in this process. Some studies have also reported that the addition of biochar in the soil regulates its physicochemical properties, such as pH, redox potential, cation exchange capacity (CEC), and organic composition [29, 30]. Additionally, biochar reduces the bioavailability of metal(loid)s in the soil, their uptake by plants, and their potential hazards to agricultural ecosystems [31]. Numerous recently published reports mainly focus on biochar-based phytoremediation in various field crops [32–34]. However, only a few studies have focused on the potential of biochar in metal remediation in tobacco crops. In this review-based study, we comprehensively evaluated the toxic effects of heavy metals on tobacco plants at physiological, biochemical, and molecular levels. We also discussed and compared numerous techniques used for the remediation of heavy metals in tobacco plants, with particularly attention to the potential of biochar application for remediating heavy metal-contaminated soils.

## 2. Search Strategy

This review-based study involved a comprehensive search of published studies conducted across various electronic databases, including PubMed, Scopus, Web of Science, Google Scholar, and others. The search employed specific keywords and corresponding terms in titles and/or abstracts: “biochar” AND “metal toxicity” AND “remediation” OR “heavy metal stresses.” The search results were screened by evaluating the titles, abstracts, and full texts of the papers. An initial screening phase eliminated duplicates, followed by the selection of unique articles based on title, abstract, and full-text assessment.

## 3. Heavy Metals in Tobacco Plants

Heavy metals such as cadmium, lead, and mercury pose a significant threat to plant growth, development, and the ecological environment [35, 36]. Currently, heavy metal pollution has become the main focus of academic research due to its detrimental effects on the plant–soil system [37]. Studies have shown that plants may react abnormally when they absorb heavy metals, subsequently, affecting animals and humans indirectly [38]. Prolonged exposure to these metals through crop plants can impact their normal functioning and overall health, posing a serious risk to the environment, animals, and humans. Controlling heavy metal pollution has become a significant concern in academic circles, particularly regarding environmental health and food security. According to Asaari et al. [38], plants subjected to heavy metal stress may exhibit various responses, such as dysplasia, wilt, and vegetation branching. These adverse reactions primarily lead to internal structure abnormalities, defoliation, withering, and even plant mortality, affecting overall morphogenetic processes [39]. Heavy metals are elements exhibiting metallic features with an atomic mass > 20 and specific gravity > 5 [40]. It is well documented that HMs, including transitional elements and metalloids, pose serious harm to humans, animals, and plants [41]. These elements, known for their toxicity even at low concentrations, are common examples of harmful HMs [41]. In nature, there are 53 elements classified as HMs, which are typically not involved in plant metabolism [42].

HMs have long been utilized by humans in the production of alloys, metals, cement, rubber, paper, and other materials [43]. Additionally, certain HMs, such as iron (Fe), copper (Cu), cobalt (Co), zinc (Zn), and molybdenum (Mo), serve as essential micronutrients in metabolic reactions [44]. However, their behavior is considered hazardous to plant development when their concentrations exceed the threshold, as outlined in Table 1. Density is the primary criterion used to categorize heavy metals [55, 56]. Other metallic elements, including aluminum (Al), antimony (Sb), mercury (Hg), and nickel (Ni), have also been investigated for their adverse effects on plants at elevated concentrations. This is especially true for toxic elements such as arsenic (As), cadmium (Cd), lead (Pb), and chromium (Cr) [56].

**Figure 2:**
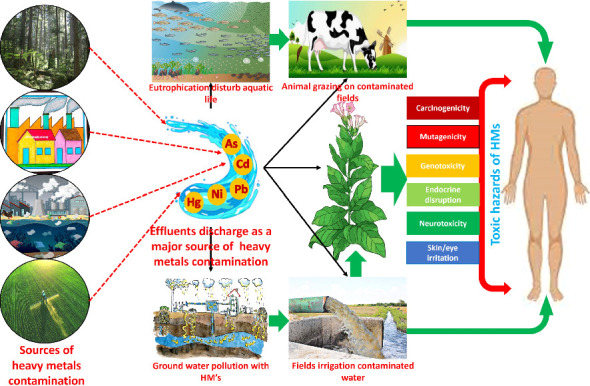
Sources of heavy metal contamination and its effect on aquatic, animal, and tobacco plants disturb human health.

Aluminum toxicity in plants, for instance, is associated with the global increase in acidic soils (40% of the world's arable land). The most toxic form of aluminum, Al^3+^, becomes available at acidic pH levels [57]. Previously, it has been reported that interactions of HMs with sulfhydryl groups can lead to impaired protein function and the upregulation of oxidative stress in crop plants [58]. In tobacco plants, soil Cd is primarily absorbed through the roots and translocated to the branches, resulting in substantial Cd accumulation in cigarette shoots [59]. At the cellular and molecular levels, heavy metal contamination causes various physiological disruptions, including the inactivation and denaturation of enzymes and proteins, as well as the blocking of functional groups in metabolically important molecules. Additionally, heavy metals can displace or replace essential metal ions in biological molecules and cellular structures, causing conformational changes and disrupting membrane integrity [60]. These changes ultimately lead to alterations in plant metabolic processes, including inhibited photosynthesis, respiration, and the activities of numerous essential enzymes [61, 62]. Moreover, these metals disrupt redox homeostasis by provoking the formation of free radicals and reactive oxygen species (ROS). Some recent studies have also reported increased production of cytotoxic compounds such as methylglyoxal under heavy metal stresses [63]. The elevated production of these cytotoxic compounds facilitates ROS accumulation in plant cells by interfering with physiological and metabolic events, including impaired antioxidative defense mechanisms, reduced photosynthesis, and respiration [64]. The excessive accumulation of ROS and high production of methylglyoxal in cells also result in lipid peroxidation, degradation of biological macromolecules, membrane dismantlement, ion leakage, and DNA strand cleavage, ultimately leading to plant death (Figure 2). In conclusion, while some HMs at low concentrations can be beneficial to plants, most are toxic to tobacco plants, causing numerous disorders in plant functioning.

## 4. Health Hazards of HMs Containing Tobacco and Its Impact on the Environment

According to the World Health Organization (WHO), global cigarette consumption has increased significantly in recent years. Due to this prevalence of smoking, tobacco and its products contribute substantially to morbidity and mortality rates. The leading cause of mortality and primary cause of illness is the high concentration of carcinogenic HMs in soil and tobacco plants. Chromium, As, Cd, Pb, and Ni are some of the carcinogens found in cigarettes with their concentrations varying based on factors such as the use of organic and inorganic fertilizers, cultivation methods, the cultivars used, and geographical location [65]. The use of tobacco and its products containing carcinogenic heavy metals increases the risk of cancer, stroke, lung and heart diseases in smokers, as well as second-hand and third-hand smokers, while also contributing to the environmental pollution [66]. The continuous release of various tobacco smoke pollutants contaminates the air annually, with indoor air being particularly affected by health-hazardous tobacco emissions [67].

## 5. Techniques Used to Detect HMs

Due to their pervasive presence as environmental pollutants, heavy metals pose growing ecological, nutritional, and environmental concerns. As a result, biomonitoring techniques are gaining popularity for their ability to predict, identify, and mitigate potential environmental risks caused by heavy metal pollution [68]. Many analytical techniques are widely used to detect HMs in plants, including atomic spectroscopy, fluorescence spectroscopy, atomic emission spectroscopy, neutron activation analysis, and anodic stripping voltammetry [69]. For instance, a study in Ghana utilized AAS to detect Fe, Cd, Pb, and Zn in various medicinal plants [69]. These biomonitoring approaches play a crucial role in predicting, recognizing, and reducing potential environmental concerns associated with heavy metal contamination (Table 2).

Furthermore, the heavy metal concentrations obtained from a field portable x-ray fluorescence (XRF) spectrometer were validated through inductively coupled plasma-mass spectrometer [80].

### 5.1. Flame Atomic Absorption Spectrometers (FAAS)

Copper and manganese levels in herbs can be effectively measured using FAAS [81]. Moreover, another study used FAAS to measure Pb levels in a Chinese herb, achieving enhanced precision after sample separation and enrichment [82]. Inductively coupled plasma optical emission spectroscopy (ICP-OES) is an advanced technique for detecting HMs and other compounds in plant materials. Recently, ICP-OES was used to detect HMs in some desert plants, yielding significant findings [45].

In FAAS, the flame serves as the energy source, typically utilizing an air-acetylene mixture for phytochemical analysis and heavy metal detection [71]. For example, FAAS was used to analyze four parts of a herbal plant for HM bioaccumulation. The findings revealed significant levels of Rb, Mn, Sb, and Sc in various parts of the plant. However, toxic HMs, including As and Hg were detected in concentrations exceeding 1 mg/g, while Cd and Pb were found at levels of 5 mg/g and 10 mg/g, respectively [83].

On the other hand, GFAAS is used to assess the bioaccumulation of HMs in solid plant samples. This method offers a time- and labor-saving alternative to traditional acid digestion techniques, due to its enhanced analytical sensitivity and graphite furnace electrothermal evaporation injection [84]. Researchers have successfully applied GFAAS to medicinal plants, yielding positive results. Heavy metals such as Hg, Pb, and Cd were analyzed in herbs like *Bombax ceiba* and *Zingiber officinale Roscoe* [72, 85].

### 5.2. The XRF Spectrometry

In the late 1990s, researchers utilized XRF spectroscopy to examine natural specimens, drawing significant conclusions about their composition [86]. XRF provides comprehensive information on substance composition, which is beneficial for population health surveys. The technique enables multi-element analysis with excellent sensitivity, particularly in soil element characterization, and produces highly accurate results [87]. XRF spectroscopy has become a standard method for detecting heavy metals in plants. It has proven effective in rapidly determining concentrations of Fe, Ti, and Mn in soil, as well as identifying the distribution of chemical elements in different parts of the plant [88]. XRF spectrometry is employed without destroying the natural specimens [86]. One of the key advantages of XRF is its nondestructive nature, which preserves the integrity of natural specimens. This method is widely used to measure heavy metals such as Fe, Ti, Mn, Cr, Cu, and Ba in plants and soils, offering precision, accuracy, and sensitivity. XRF can also detect the allocation of elements in various sections of the plant, including the rhizome, stem, leaves, and flowers [88].

### 5.3. Inductively Coupled Plasma Mass Spectrometry (ICP-MS)

In addition to the promising detecting technologies mentioned earlier, one of the latest techniques involves using ICP-MS to analyze the absorption of HMs and other biological macromolecules. This method, utilizing ICP-MS for the absorption of HMs and other biological macromolecules, is among the most recent analytical approaches, complementing the array of excellent methods available [45]. ICP-MS spectroscopy is considered the gold standard for determining the metal content of medicinal plants, due to its ability to rapidly detect minor variations, simultaneously measure multiple elements, and offer high accuracy. Moreover, ICP-MS can be employed for multielement trace and isotope analyses, making it a powerful tool for elemental analysis [89]. It is often combined with other analytical techniques to improve the detection of heavy metals in medicinal plants. In a previous study, a researcher utilized ICP-MS and electrospray ionization mass spectrometry to examine the elements in dried *Lycium barbarum.* The detection limits for the analyzed elements were as follows: Mn, 0.42 g/L; Cu, 0.22 g/L; and Zn, 1.6 g/L [90]. Several authors have proposed various methods to monitor the concentrations of heavy metals in plants, among which AAS and atomic fluorescence spectrum (AFS) being commonly used. However, these technologies have limitations such as low efficiency and high price. Therefore, some researchers have suggested ICP-OES, XRFS, and other methods for heavy metals detection in plants. However, these techniques require a chemical laboratory and expensive equipment as well and are often time-consuming, complex, and costly [50, 91].

## 6. Removal and Remediation Techniques of HMs

World's food supply has been threatened by heavy metal stress in plants, rising significant concerns among scientists globally. Experts are increasingly aware of the dangers posed by heavy metal stress and have taken immediate steps to mitigate its effects on plant health [92]. Since their density exceeds 5 g/cm^3^, 53 of the 90 naturally occurring elements are classified as HMs [93]. Plants require 17 metals to thrive, which are classified as micronutrients. However, when present in excess, these micronutrients can negatively affect plant growth and survival [74]. Another group of elements, including Hg, Ag, Cd, Pb, Cr, and aluminum (Al), can elicit HM toxicity when absorbed by the plant's metabolism, as they provide no nutritional value [94]. In recent decades, rapid industrialization, modern farming methods, and increased anthropogenic activities have all contributed to a dramatic increase in the accumulation of HMs. These modern farming techniques and industrial processes have significantly enhanced the buildup of HMs in the environment [95], especially in tobacco plants, which are the most used plants worldwide [96]. HMs enter plants through their roots, adversely affecting processes such as photosynthesis, mineral uptake, and water absorption [97]. The accumulation of HMs in tobacco plants has reached alarming levels, making it crucial to address this issue to protect both tobacco plants and human health from the toxic effects of HMs. Various biological and chemical techniques have been employed to reduce the toxicity of HMs in contaminated soil, water, and plants. For instance, cementation, adsorption [98], ion exchange, filtration [99], solvent extraction, soil washing [100], stabilization, excavation, and other methods can be utilized for remediating HMs [101]. Chemical precipitation is also among the useful techniques in this regard [101].

For instance, recent research has highlighted auxin's ability to enhance plant stress resistance by reducing absorption, increasing chelation and vacuolar sequestration, and minimizing stress-induced oxidative damage. Additionally, auxin interacts with other biomolecules such as NO, CO, ethylene, and abscisic acid, thereby protecting plants from oxidative stress induced by various challenging conditions. Auxin might contribute to plant resilience to stress through mechanisms such as decreased absorption, increased chelation and sequestration in plant organelles, and reduced oxidative lesions associated with stress [102]. In the context of heavy metal removal, biological techniques targeting key environmental components such as soil, air, water, and plants can be employed. Both active (metabolic and energy-dependent) and passive bioremediation procedures can utilize either dead or living organisms/biomass. In positive (metabolic and energy-dependent) or negative bioremediation techniques, it is feasible to use either deceased or living wage organisms/biomass. Microorganisms can be directly applied to contaminated areas or utilized in bioreactors optimized for cleanup purposes. Phytoremediation involves the use of plants to cleanup polluted areas, either by growing them on contaminated soil or utilizing wetland biomass. This chapter introduces the usage of plants in biological heavy metal remediation. Microorganisms employ various techniques such as biosorption, biotransformation, bioaccumulation, bioleaching, and biomineralization during bioremediation [103]. Although bioremediation is reported to be a highly eco-friendly and cost-effective approach for metal remediation, some studies have also discussed its downsides. Since the process of microbial degradation is slower, bioremediation can take longer than other remediation approaches [104]. Moreover, this method is not effective for all types of pollutants. Another strategy for HM removal in plants involves “genetic engineering techniques,” which enhance plant tolerance and toxic metal accumulation, greatly aiding phytoremediation. Recent studies have utilized omics methods such as proteomics and metabolomics to gain a better understanding of the genetic determinants and pathways associated with HM stress tolerance in plants. HMs and metalloids such as Hg, Cd, Se, and As are being removed from the environment by plants using biotechnological methods. Currently, three major biotechnological methods are being employed to engineer plants for heavy metal and metalloid phytoremediation. Using biotechnological techniques, plants remove HMs and metalloids from the soil, including Hg, Cd, Pb, Se, and As. These three biotechnological methods are extensively utilized for modifying plants to enhance their capacity for heavy metal and metalloid phytoremediation [105].

Auxin, a plant hormone crucial for development and growth, plays a pivotal role in regulating the plant's response to environmental stresses by overseeing biosynthesis, conjugation, and degradation processes [106, 107]. It is internationally recognized for its role in modulating both normal and stressed root growth in plants. Given that roots are the primary organs responsible for detecting heavy metal stress, understanding the interplay between heavy metal stress and auxin homeostasis is of paramount importance. In addition to its well-known role in regulating healthy and stressed root growth in plants, auxin has gained international recognition as a key player among other phytohormones. Understanding how HM stress stimuli interact with auxin homeostasis is crucial, given that roots are the primary organs responsible for detecting HM stress [108]. HMs alter auxin homeostasis by influencing auxin concentration and distribution within the plant. Several studies have observed variations in endogenous auxin levels in root and shoot tissues following exposure to HM stress. Additionally, a complex relationship exists between auxin concentrations and heavy metal toxicity, which can be both positive and negative [109].

**Figure 3:**
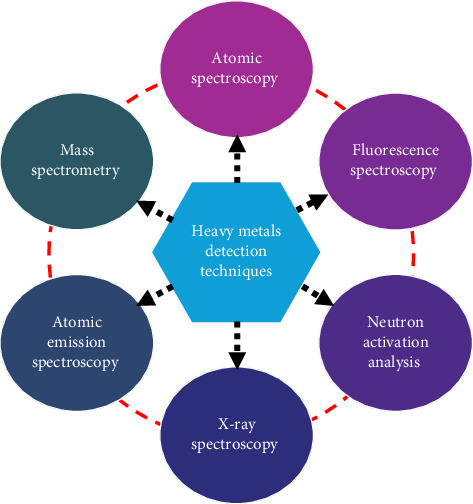
The techniques used in heavy metals detection in vegetation plants and soil.

Phytoremediation is a process that uses plants to mitigate heavy metal toxicity in contaminated environments, addressing both organic and inorganic pollutants. Organic pollutants can be remediated through techniques such as rhizoremediation and degradation, while inorganic pollutants are typically addressed using extraction methods [110]. Phytoremediation is recognized as a feasible and cost-effective approach for environmental cleanup. The effectiveness of phytoremediation relies on various factors, including the root structure of the plant (tap or fibrous), its ability to survive and adapt to the ecological conditions, its growth rate at the specific site, and its resistance to pests and diseases. Certain plants, known as hyperaccumulators, have the capability to absorb metals, transport them through the xylem, and accumulate them in their shoots, thereby reducing pollution [111]. During phytoremediation, metals are not entirely eliminated but rather transferred from one system to another. In phytostabilization, metals are absorbed and stored in the root system, reducing soil contamination. This process prevents hazardous metals from leaching into the soil and water supply, thus protecting the food web [112]. Despite the effectiveness of phytoremediation, there are several limitations in removing heavy metals from plants. This approach is often considered a slow process, requiring multiple growing seasons to achieve significant contaminant reduction. Additionally, since plant roots typically develop in the upper soil layers, phytoremediation is reported to be less effective for deeply contaminated sites [50]. Furthermore, factors such as plant health, growth rate, and environmental conditions—like soil fertility, pH, and climate—can significantly influence the effectiveness of phytoremediation [113]. Therefore, developing novel techniques for the remediation of heavy metals in plants is warranted (Figure 3).

## 7. Biochar as Remediation Strategy for the Removal of HMs in Plant

**Figure 4:**
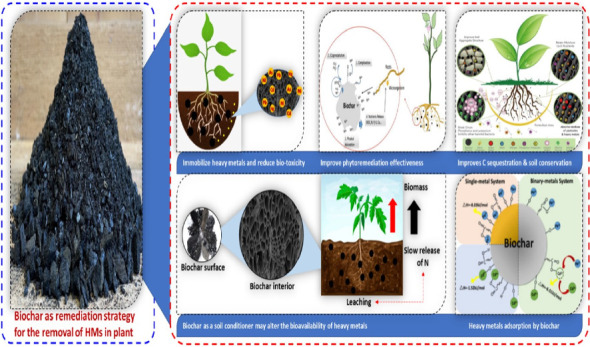
The use of biochar for the reduction of heavy metals in plants to improve soil fertility and plant growth and production by alleviating heavy metals stress.

Biochar possesses unique physicochemical properties, including a porous structure, a significant exposed surface area, and an abundance of surface functional groups, making it promising environmental amendment. It is produced as a carbonaceous byproduct when biomass undergoes pyrolysis in an oxygen-limited or oxygen-free environment [114]. The aromatic C structure of chars, including biochar, comprises both an amorphous phase with disorganized aromatic rings and a crystalline phase with condensed polyaromatic sheets. This distinction in aromatic carbon structure differentiates chars, such as biochar [115]. Reports indicate that biochar usage can immobilize environmental heavy metals, thereby reducing their toxicity to organisms. The utilization of biochar has been shown to immobilize environmental HMs, thereby reducing their toxicity to living organisms [116]. For example, metals such as Cd and Pb can be immobilized in biochar through complexation, as demonstrated by Xu et al. who found that bamboo biochar has the ability to immobilize Cd and Pb [117]. This suggests that biochar has the potential to decrease the levels of Cd and Pb in soil by immobilizing these metals through complexation and ion conversion [117]. Biochar has the capability to modify the behavior of HMs in contaminated sites even in the absence of plants [118]. In a field experiment, the bioavailability of Cd, Cu, Pb, and Hg was investigated using pak choi as the test plant. The incorporation of biochar into the soil significantly increased crop yield. Application of biochar at various rates (ranging from 0 to 1.5 to 2.25 to 3.0 t·ha^−1^) resulted in a reduction in the bioavailability of HMs to plant roots. Specifically, the bioavailability of HMs in plant roots decreased with increasing rates of biochar application. This study suggests that sugarcane bagasse biochar has the potential to remediate polluted soils, thereby enhancing both crop yield and quality [118]. The cultivation of heavy metals has led to the accumulation of numerous contaminants that must be addressed for soil remediation. HMs in soil and plants may exhibit different behaviors following interactions with biochar, plants, and microorganisms. Heavy metal accumulation has led to the accumulation of a wide range of toxins that require remediation from the environment. Interaction with biochar, plants, and microbes can elicit varied responses from heavy metals in both the environment and plants. The ability of biochar to enhance plant growth and microbial activity could potentially enhance the effectiveness of phytoremediation [119]. However, there has been limited research on the impact of biochar on heavy metal behavior, plant toxicity, and microbial activity in sediments where plants thrive. To address this gap, the functions of biochar derived from tea waste were investigated by growing seedlings of *Boehmeria nivea* (L.). Tea waste, as a form of biomass, poses disposal challenges, but it offers a practical and cost-effective approach for environmental contaminant removal ([120]; Figure 4).

### 7.1. Biochar–Plant–Soil Interactions for Metals Remediation

Biochar, also referred to as biocharcoal or biocoal, is produced through the gradual thermal decomposition of biomass at high temperatures and low oxygen levels. This process yields a type of black carbon with several beneficial properties, including high stability and adsorption capacity, significant aromatization, and high porosity. Biochar is characterized by its high carbon content, which remains thermally and biochemically stable over time [33]. The application of biochar offers advantages in both carbon sequestration and soil conservation. In a previous study, the potential of biochar to enhance tobacco plant growth was investigated through a 3-year field experiment. Various levels of biochar were applied, and their effects on the growth indices of tobacco plants were assessed. Throughout the growth period, root development, chlorophyll content, and leaf-area parameters remained consistent. Compared to control plants, those treated with biochar exhibited a notable increase in root vitality, up to 177.8%. Moreover, biochar application led to a significant enhancement in root area (+91.35%) and the number of root tips number (+100.9%). Additionally, biochar-treated tobacco plants showed a substantial increase in net photosynthetic rate, up to 77.3% higher than control plants [33].

In addition to improving soil fertility, the use of biochar as a soil conditioner can potentially alter the bioavailability of HMs, which may have significant implications for the environment. Previous research conducted a 2-year field experiment in southwestern China, using tobacco plants in yellow-brown soil (pH 5.32), to investigate the effects of biochar on HM bioavailability in soils. The results revealed significant increases in Cd levels in acidic soil when biochar was applied at a rate of 40 Mg·ha^−1^, whereas only a slight increase was observed in neutral yellow soil at a rate of 15 Mg·ha^−1^. However, the addition of biochar did not lead to a significant increase in Cd content or accumulation in crops. The duration after biochar application and soil pH also influenced the decline in accessible Cu, Ni, and Pb. Furthermore, increasing the biochar application rate resulted in a decrease in the amount of Cu, Ni, and Pb in tobacco plants. While the high Cd content in biochar contributed to increases in soil-available Cd, the decreases in soil-available Cu, Ni, and Pb levels could be attributed to their immobilization through processes such as ion exchange and complexation ([34]; Table 3).

In a previous study, the efficiency of tobacco plants in absorbing Cd was examined using biochar derived from corn cobs and rice husks [125]. The findings revealed that the concentration of Cd absorbed by tobacco plants, especially in the shoots, increased with higher Cd dosages (*p* < 0.01). Interestingly, despite the overall increase in Cd levels and contents, treatments involving corn cob biochar produced at various pyrolysis temperatures led to decreased shoot Cd levels (*p* < 0.01). Conversely, treatments with rice husk biochar did not exhibit the same effect. The study concluded that tobacco plants benefited more from treatments with corn cob biochar than those with rice husk biochar, particularly in terms of reducing Cd uptake [126].

### 7.2. Mechanisms of Biochar Interaction With Cadmium and Other Heavy Metals

In recent years, extensive research into biochar's potential as a soil amendment has revealed its numerous positive effects when applied to agricultural soils. For example, biochar has been shown to reduce soil bulk density, increase soil nitrogen levels, and enhance soil organic carbon levels [127]. Additionally, studies have demonstrated its efficacy in remediating HMs and plant growth [34, 128]. However, the exact mechanism behind the fixation of metal ions by biochar is not fully understood. Some recent studies however reported heavy metal fixation by biochar application through ionic exchange, coprecipitation, cation *π*-electron interaction, and complexation [34, 129]. However, the adsorption capacity of biochar is highly dependent on its properties and pyrolysis conditions. According to Cui et al. [130]; mineral precipitation and ion exchange mainly occurred during metal remediations under biochar application. At plant level, biochar application has been reported to improve growth, development, physiology, yield, and overall productivity of tobacco crop. Adsorption, the major mechanism through which biochar interacts with metal ions, primarily occurs through complex interactions between the metal ions and the biochar surface [131]. The highly porous structure, with micro- and macropores, and the large surface area of biochar provide numerous sites for the adsorption of metal ions [132]. These sites also reduce the bioavailability of metal ions in the environment. Additionally, electrostatic adsorption onto the biochar surface is another important phenomenon in metal remediation. Several studies have reported that biochar surfaces often possess a higher negative charge, which attracts positively charged metal ions [133]. Pyrolysis conditions, including temperature and environmental pH, can influence these electrostatic interactions. It is commonly believed that higher pyrolysis temperatures and pH conditions enhance the negative charge, thereby increasing the metal adsorption capacity. Furthermore, metal remediation through biochar is also mediated by physical adsorption [134]. Van der Waals forces contribute to the physical adsorption of metal ions on the biochar surface; however, physical adsorption is generally considered less effective than chemical adsorption.

#### 7.2.1. Physical and Chemical Properties of Biochar Affecting Its Remediation Capability

Biochar's surface area and porosity are the major physical properties that affect its effectiveness in the remediation of metal-contaminated sites [135]. Biochar is largely characterized by its highly porous structure, which develops during the pyrolysis process. Various published reports have documented that a large surface area provides more adsorption sites for metal ion interactions [136]. Moreover, the adsorption capacity of biochar is directly influenced by its porous structure, with the porosity being essential for trapping metal ions [137]. For physical adsorption, micropores offer high surface areas, whereas mesopores and macropores play crucial roles in the diffusion and movement of metal ions. Some studies have also noted that the pore size distribution can affect the metal adsorption rates [137]. In addition to surface area and particle size, the structural stability and durability of biochar influence its long-term effectiveness in metal ion remediation. Due to its high carbon content, biochar is reported to be more stable than other substances used in remediation [138]. Moreover, its high resistance to microbial degradation makes it a more suitable and sustainable option for remediation.

Furthermore, some chemical properties of biochar also influence its effectiveness in metal remediation. The chemical properties, including the presence of surface functional groups, are crucial in determining the metal adsorption capacity of biochar [139]. During the pyrolysis process, functional groups such as negatively charged carboxyl (-COOH), hydroxyl (-OH), phenolic, and carbonyl (-C=O) provide numerous active sites for metal attachment through mechanisms such as complexation, ion exchange, and electrostatic attraction. In addition to these groups, the CEC and pH buffering capacity of biochar also determine its ability to provide charges on its surface for metal ion adsorption [140]. High CEC values are typically attributed to the high presence of negatively charged functional groups, enhancing the adsorption of positively charged metal ions such as Cd^2+^, Pb^2+^, and Zn^2+^. Moreover, the environmental pH is crucial in determining biochar's adsorption capacity. It is well established that biochar produced under high pyrolysis temperature conditions tends to be more alkaline due to the presence of ash and inorganic minerals [141]. This alkaline nature helps neutralize acidic soils, raise the pH, and increase the precipitation of metal hydroxides. Higher pH is also reported to boost the adsorption of heavy metals [136]. Overall, biochar has emerged as a promising solution for the remediation of contaminated sites. Its effectiveness in metal adsorption is significantly influenced by factors such as high surface area, porous structure, and the presence of functional groups such as carboxyl (-COOH) and hydroxyl (-OH). Under biochar application, metal adsorption primarily occurs through physical adsorption, where metal ions are trapped in the pores of the biochar. Additionally, electrostatic attraction and ion exchange are commonly reported mechanisms of metal remediation facilitated by biochar.

## 8. Conclusions

This review study summarizes the effects of HMs on tobacco plants, noting that HMs primarily accumulate in the roots, followed by stems, barks, and leaves. Various analytical techniques, including AAS, FAAS, GFAAS, CVAAS, ICP-OES, and ICP-MS, are utilized to detect HM bioaccumulation in plants. Studies have revealed significant effects of HMs on plants, such as reduced growth, increased oxidative stress, and inhibited seed germination, necessitating effective solutions. To address HM-related risks in plants, various methods have been employed, with biochar emerging as a promising solution due to its distinct physicochemical properties, such as a large surface area and numerous surface functional groups. Corn cob biochar treatments, in particular, show promise in mitigating HM toxicity in plants. Integrating biochar with advanced technologies and understanding the plant-soil-microbe nexus opens new avenues for sustainable agriculture and environmental remediation. This review article advocates for further research in this field to unlock the potential of biochar as a game-changer in addressing metal stress and promoting the resilient cultivation of tobacco plants. With biochar as the compass, the journey into tobacco's heavy metal voyage aims for sustainable agricultural crescendos.

## Figures and Tables

**Table 1:** The bioaccumulation of various HMs in different plant species and their effects.

Plant name	Detected HMs	Translocation	Obtained findings	Reference
*Tetraena qataranse*	Ba, Cd, Cr, Cu, Ni, and Pb	Detected in biomass specially in roots	*T. qataranse* was tolerant of Cd, Cr, Cu, and Ni but was susceptible to both Pb and Ba	[45]
Tomato	Cr	Roots, shoot, and tomato fruit	Germination and seedling growth were found be to very sensitive toward 200–400 mg/kg of Cr	[46]
Cumin	Ni	Seeds and leaves	Chlorophyll a and b were significantly decreased	[47]
Tobacco	Ba, Br, Ca, Cd, Rb, Sb, and Yb	Tobacco leaves	Rubidium and Cd were found in higher concentrations which could be a possible pose health hazard	[48]
Transgenic tobacco (*Lobularia maritima*)	Cd, Cu, and Zn	Seedlings	Plants showed higher tolerance to Cd and Mn than Cu and Zn	[49]
Tobacco	Hg	Leaves	An increase in Hg concentration was inversely proportional to the seedling growth	[50]
Sunflower	Cd and Pb	—	Growth was inhibited. However, the presence of Cd and Pb enhanced the biosynthesis of thiol group	[51]
Wheat	Cd	Root, shoot, and Grains	A dose (10 mg/kg) of Cd is found hazardous for all the physio-morphological traits of wheat	[52]
*Artemisia campestris*	Cd, Ni, Cr, Mn, Co, and Fe	Leaves	Ni (53.64 μg/g) was found to be the most abundant among all HMs	[53]
Herbaceous plants (*Combretum mucronatum*)	Cr, Zn, Cu, and Cd	Leaves and roots	The significant bioaccumulation of HMs and reduced plant growth	[54]

**Table 2:** Various analytical techniques for the detection of heavy metals in plants.

Analytical technique	Obtained findings	Reference
Atomic absorption spectrometer	This technique is commonly recommended to detect HMs in medicinal plants. The analytical technique exhibited good performance where the lead concentration was higher 3.37 microg/g in dry plant ash	[70]
Flame atomic absorption spectrometer	Native Amazonian fruit was used as model for the absorption of HMs. HMs were detected in varies form Ca (402–8750 mg/kg) and Mg (359–2198 mg/kg)	[71]
Graphite furnace atomic absorption spectrometer (GFAAS)	The flowers and fruits of *Lonicera tatarica* were used to detect the HMs. Mostly, Zn, Cr, Cd, Pb, and Cu were significantly detected in the flowers	[72]
Atomic absorption spectrometer	Stem, leaf, and bark of the neem plant (*Azadirachta indica*) were used as model specie for the bioaccumulation of HMs. The findings revealed that Cr, Pb, Cu, Ni, and Cd were significantly abundant in stem as compared to bark and leaves	[73]
Cold vapor atomic absorption spectrometer (CVAAS)	Aloe vera, *Centella asiatica,* and *Cucumis sativus* were used to detect the HMs. The HMs were found within the prescribed limit. However, other trace elements were found in abundant	[74]
High pressure digestion-hydride generation atomic fluorescence spectrometer	*Cordyceps sinensis* was used as a major phyto species	[75]
Inductively coupled plasma atomic emission spectrometry (ICP-AES)	The technique efficiently detected Ca, Cd, Cr, Ni, and Pb in the root, rhizome, seed, gall, and fruits of *Cichorium intybus*	[76]
Inductively coupled plasma mass spectrometry	The barks of *Cortex salicis* for Cd and Pb. Due to the limited extraction efficiency of this metal, there was little cadmium in the infusions, making them a relatively safe herbal medication	[77]
XRF	Examined seed and leaf of *Anisum vulgare*. The X-ray fluorescence analyzed with synchrotron radiation can be used to identify the elemental profile of medicinal plants. It is fast, sensitive, robust, multi-elemental, and high throughput	[78]
HPLC	*Angelica sinensis* was analyzed for HMs. Pb, Cu, Hg, and other metals were detected in different parts of the plant	[79]

**Table 3:** The interaction of heavy metals (HMs) and biochar for the removal of these metals.

Metal/s	Biochar source	Effect	Reference
Cadmium and copper	Dairy manure	Cadmium is prone to an absorption process through cation exchange, which increases with rising pH levels	Xu et al. [121]
Lead	Sludge-derived biochar	Lead is primarily absorbed through precipitation. Increasing temperature promotes the sorption of this metal, which is mainly associated with the mineral composition of the biochar used	Lu et al. [28]
Mercury	Soybean stalk-based biochar	The major mechanisms for the sorption of this metal are ion exchange, complexation, and precipitation. However, these mechanisms are highly influenced by biochar characteristics and pyrolysis conditions	Kong et al. [122]
Arsenic (As(III) and As(V))	Empty fruit bunch and rice husk	The complexation and chemical reduction are main mechanisms for the sorption of arsenic	Samsuri et al. [123]
Chromium	—	When biochar interacts with Cr, it supports the transformation from Cr(VI) to Cr(III). In this process, biochar offers a suitable number of functional groups that can be obtained with suitable feedstock and slow pyrolysis	Li et al. [124]

## Data Availability

The datasets generated during and/or analyzed during the current study are available from the corresponding author on reasonable request.

## References

[1] Tong Z., Xu M., Zhang Q. (2023). Construction of a High-Density Genetic Map and Dissection of Genetic Architecture of Six Agronomic Traits in Tobacco (*Nicotiana tabacum* L.). *Frontiers in Plant Science*.

[2] Sirotkin A. V., Kolesarova A. (2022). *Environmental Contaminants and Medicinal Plants Action on Female Reproduction*.

[3] Goncharuk E. A., Zagoskina N. V. (2023). Heavy Metals, Their Phytotoxicity, and the Role of Phenolic Antioxidants in Plant Stress Responses with Focus on Cadmium: Review. *Molecules*.

[4] Rai P. K., Sonne C., Kim K. H. (2023). Heavy Metals and Arsenic Stress in Food Crops: Elucidating Antioxidative Defense Mechanisms in Hyperaccumulators for Food Security, Agricultural Sustainability, and Human Health. *The Science of the Total Environment*.

[5] Regassa G., Chandravanshi B. S. (2016). Levels of Heavy Metals in the Raw and Processed Ethiopian Tobacco Leaves. *SpringerPlus*.

[6] Verma S., Yadav S., Singh I. (2010). Trace Metal Concentration in Different Indian Tobacco Products and Related Health Implications. *Food and Chemical Toxicology*.

[7] Golia E. E., Dimirkou A., Mitsios I. K. (2008). Levels of Heavy Metals Pollution in Different Types of Soil of Central Greece. *Bulletin of Environmental Contamination and Toxicology*.

[8] Lugon-Moulin N., Ryan L., Donini P., Rossi L. (2006). Cadmium Content of Phosphate Fertilizers Used for Tobacco Production. *Agronomy for Sustainable Development*.

[9] Semu E., Singh B. R. (1995). Accumulation of Heavy Metals in Soils and Plants After Long-Term Use of Fertilizers and Fungicides in Tanzania. *Fertilizer Research*.

[10] Naz M., Hussain S., Ashraf I., Farooq M. (2022). Exogenous Application of Proline and Phosphorus Help Improving Maize Performance Under Salt Stress. *Journal of Plant Nutrition*.

[11] Sarmah R., Sarma A. K. (2023). Phosphate Solubilizing Microorganisms: A Review. *Communications in Soil Science and Plant Analysis*.

[12] Karaivazoglou N. A., Tsotsolis N. C., Tsadilas C. D. (2007). Influence of Liming and Form of Nitrogen Fertilizer on Nutrient Uptake, Growth, Yield, and Quality of Virginia (Flue-Cured) Tobacco. *Field Crops Research*.

[13] Engida A. M., Chandravanshi B. S. (2017). Assessment of Heavy Metals in Tobacco of Cigarettes Commonly Sold in Ethiopia. *Cosmetics International*.

[14] Pieruschka R., Schurr U. (2019). Plant Phenotyping: Past, Present, and Future. *Plant Phenomics*.

[15] Wang J., Hussain S., Sun X. (2022). Nitrogen Application at a Lower Rate Reduce Net Field Global Warming Potential and Greenhouse Gas Intensity in Winter Wheat Grown in Semi-Arid Region of the Loess Plateau. *Field Crops Research*.

[16] Wang Y. C., Ni J. J. (2023). Biochar Application on Heavy Metal Immobilization in Unsaturated Soil with Vegetation: A Review. *International Journal of Geotechnical Engineering*.

[17] Melkamu T., Abera G., Asere T. G. (2022). Determination of Heavy Metals in Tobacco Leaves and Their Growing Soils in Assosa District, Benshangul Gumuz Regional State, Ethiopia. *Journal of the Turkish Chemical Society Section A: Chemistry*.

[18] Li Y., Ahlstrom D. (2020). Risk-taking in Entrepreneurial Decision-Making: A Dynamic Model of Venture Decision. *Asia Pacific Journal of Management*.

[19] Knickel K., Redman M., Darnhofer I. (2018). Between Aspirations and Reality: Making Farming, Food Systems and Rural Areas More Resilient, Sustainable and Equitable. *Journal of Rural Studies*.

[20] Thilakan D., Patankar J., Khadtare S. (2022). Plant‐Derived Iron Nanoparticles for Removal of Heavy Metals. *International Journal of Chemical Engineering*.

[21] Zhang L., Ruiz-Menjivar J., Luo B., Liang Z., Swisher M. E. (2020). Predicting Climate Change Mitigation and Adaptation Behaviors in Agricultural Production: A Comparison of the Theory of Planned Behavior and the Value-Belief-Norm Theory. *Journal of Environmental Psychology*.

[22] Aziz H., Murtaza G., Saleem M. H. (2021). Alleviation of Chlorpyrifos Toxicity in Maize (*Zea mays* L.) by Reducing its Uptake and Oxidative Stress in Response to Soil-Applied Compost and Biochar Amendments. *Plants*.

[23] Elkhlifi Z., Iftikhar J., Sarraf M. (2023). Potential Role of Biochar on Capturing Soil Nutrients, Carbon Sequestration and Managing Environmental Challenges: A Review. *Sustainability*.

[24] El-Tohory S., Zeng W., Huang J. (2023). Effect of Intercropping and Biochar Application on Cadmium Removal Capacity by *Corchorus olitorius* and *Zea mays*. *Environmental Technology & Innovation*.

[25] Irshad S., Xie Z., Kamran M. (2021). Biochar Composite With Microbes Enhanced Arsenic Biosorption and Phytoextraction by Typha Latifolia in Hybrid Vertical Subsurface Flow Constructed Wetland. *Environmental Pollution*.

[26] Kamal A., Saleem M. H., Alshaya H., Okla M. K., Chaudhary H. J., Munis M. F. H. (2022). Ball-Milled Synthesis of Maize Biochar-ZnO Nanocomposite (MB-ZnO) and Estimation of its Photocatalytic Ability Against Different Organic and Inorganic Pollutants. *Journal of Saudi Chemical Society*.

[27] Tan X., Liu Y., Zeng G. (2015). Application of Biochar for the Removal of Pollutants from Aqueous Solutions. *Chemosphere*.

[28] Lu H., Zhang W., Yang Y., Huang X., Wang S., Qiu R. (2012). Relative Distribution of Pb2+ Sorption Mechanisms by Sludge-Derived Biochar. *Water Research*.

[29] Rehman M., Liu L., Bashir S. (2019). Influence of Rice Straw Biochar on Growth, Antioxidant Capacity and Copper Uptake in Ramie (*Boehmeria nivea* L.) Grown as Forage in Aged Copper-Contaminated Soil. *Plant Physiology and Biochemistry*.

[30] Rehman M., Saleem M. H., Fahad S. (2021). Effects of Rice Straw Biochar and Nitrogen Fertilizer on Ramie (*Boehmeria nivea* L.) Morpho-Physiological Traits, Copper Uptake and Post-Harvest Soil Characteristics, Grown in an Aged-Copper Contaminated Soil. *Journal of Plant Nutrition*.

[31] Safeer R., Liu G., Yousaf B. (2024). Insights into the Biogeochemical Transformation, Environmental Impacts and Biochar-Based Soil Decontamination of Antimony. *Environmental Research*.

[32] Guo R., Qian R., Yang L. (2022). Interactive Effects of Maize Straw-Derived Biochar and N Fertilization on Soil Bulk Density and Porosity, Maize Productivity and Nitrogen Use Efficiency in Arid Areas. *Journal of Soil Science and Plant Nutrition*.

[33] Ren T., Wang H., Yuan Y. (2021). Biochar Increases Tobacco Yield by Promoting Root Growth Based on a Three-Year Field Application. *Scientific Reports*.

[34] Zhang J., Li C., Li G., He Y., Yang J., Zhang J. (2021). Effects of Biochar on Heavy Metal Bioavailability and Uptake by Tobacco (*Nicotiana tabacum*) in Two Soils. *Agriculture, Ecosystems & Environment*.

[35] Hayat U., din K. U., Haider A. (2024). Salicylic Acid-Induced Antioxidant Defense System Alleviates Cadmium Toxicity in Wheat. *Journal of Soil Science and Plant Nutrition*.

[36] Sabir M. A., Nawaz M. F., Khan T. H. (2023). Impact of Dust Load and Lead (Pb) Stress on Leaf Functioning of Urban Vegetation. *Turkish Journal of Agriculture and Forestry*.

[37] Hussnain M., Shabaan M., Faiza (2023). Microbial Phytoremediation of Chromium-Contaminated Soil with Biogas Slurry for Enhancing the Performance of *Vigna radiata* L. *Plant Stress*.

[38] Asaari M. S. M., Mertens S., Dhondt S., Inzé D., Wuyts N., Scheunders P. (2019). Analysis of Hyperspectral Images for Detection of Drought Stress and Recovery in Maize Plants in a High-Throughput Phenotyping Platform. *Computers and Electronics in Agriculture*.

[39] Pérez-Bueno M. L., Pineda M., Barón M. (2019). Phenotyping Plant Responses to Biotic Stress by Chlorophyll Fluorescence Imaging. *Frontiers of Plant Science*.

[40] Rascio N., Navari-Izzo F. (2011). Heavy Metal Hyperaccumulating Plants: How and Why Do They Do it? and what Makes Them So Interesting?. *Plant Science*.

[41] Jamla M., Khare T., Joshi S., Patil S., Penna S., Kumar V. (2021). Omics Approaches for Understanding Heavy Metal Responses and Tolerance in Plants. *Current Plant Biology*.

[42] Rebello S., Sivaprasad M. S., Anoopkumar A. N. (2021). Cleaner Technologies to Combat Heavy Metal Toxicity. *Journal of Environmental Management*.

[43] Hussain S., Khaliq A., Noor M. A. (2020). Metal Toxicity and Nitrogen Metabolism in Plants: An Overview. *Carbon and nitrogen cycling in soil*.

[44] Angulo-Bejarano P. I., Puente-Rivera J., Cruz-Ortega R. (2021). Metal and Metalloid Toxicity in Plants: An Overview on Molecular Aspects. *Plants*.

[45] Usman K., Al-Ghouti M. A., Abu-Dieyeh M. H. (2019). The Assessment of Cadmium, Chromium, Copper, and Nickel Tolerance and Bioaccumulation by Shrub Plant Tetraena Qataranse. *Scientific Reports*.

[46] Shoaib A., Khurshid S., Javaid A. (2022). Cloncurry Buffel Grass Mitigated Cr(III) and Cr(VI) Toxicity in Tomato Plant. *Scientific Reports*.

[47] Khan N. A., Zhang R., Wang X. (2022). Assembling Covalent Organic Framework Membranes via Phase Switching for Ultrafast Molecular Transport. *Nature Communications*.

[48] El-Samad M., Hanafi H. (2017). Analysis of Toxic Heavy Metals of Cigarettes by Instrumental Neutron Activation Analysis. *Journal of Taibah University for Science*.

[49] Ben Saad R., Ben Romdhane W., Mihoubi W., Ben Hsouna A., Brini F. (2020). A Lobularia Maritima LmSAP Protein Modulates Gibberellic Acid Homeostasis via its A20 Domain under Abiotic Stress Conditions. *PLoS One*.

[50] Yu Ke-Q., Fang S., Zhao Y. (2021). Heavy Metal Hg Stress Detection in Tobacco Plant Using Hyperspectral Sensing and Data-Driven Machine Learning Methods. *Spectrochimica Acta Part A: Molecular and Biomolecular Spectroscopy*.

[51] Erhart E., Hartl W. (2009). Soil Protection through Organic Farming: A Review. *Sustainable Agriculture Reviews*.

[52] Erdem H., Tosun Y. K., Ozturk M. (2012). Effect of Cadmium-Zinc Interactions on Growth and Cd-Zn Concentration in Durum and Bread Wheats. *Fresenius Environmental Bulletin*.

[53] Melkaoui C., Cheriti A., Bouchekara M. (2023). Assessment of Heavy Metals and Macromineral in Frequently Used Medicinal Plants from Algerian Sahara Traditional Ethnopharmacopeia. *Annales Pharmaceutiques Françaises*.

[54] Bako A., Muhammad M., Isma’ila Y., Rufai M. A. (2014). *Issues on Nigerian Peoples and Culture*.

[55] Fryzova R., Pohanka M., Martinkova P. (2018). Oxidative Stress and Heavy Metals in Plants. *Reviews of Environmental Contamination & Toxicology*.

[56] Rahman Z., Singh V. P. (2019). The Relative Impact of Toxic Heavy Metals (THMs) (Arsenic (As), Cadmium (Cd), Chromium (Cr) (VI), Mercury (Hg), and Lead (Pb)) on the Total Environment: An Overview. *Environmental Monitoring and Assessment*.

[57] Von Uexküll H. R., Mutert E. (1995). Global Extent, Development and Economic Impact of Acid Soils. *Plant and Soil*.

[58] Clemens S., Ma J. F. (2016). Toxic Heavy Metal and Metalloid Accumulation in Crop Plants and Foods. *Annual Review of Plant Biology*.

[59] Hermand V., Julio E., Dorlhac de Borne F. (2014). Inactivation of Two Newly Identified Tobacco Heavy Metal ATPases Leads to Reduced Zn and Cd Accumulation in Shoots and Reduced Pollen Germination. *Metallomics*.

[60] Villiers F., Ducruix C., Hugouvieux V. (2011). Investigating the Plant Response to Cadmium Exposure by Proteomic and Metabolomic Approaches. *Proteomics*.

[61] Hossain M. A., Piyatida P., da Silva J. A. T., Fujita M. (2012). Molecular Mechanism of Heavy Metal Toxicity and Tolerance in Plants: Central Role of Glutathione in Detoxification of Reactive Oxygen Species and Methylglyoxal and in Heavy Metal Chelation. *Journal of botany*.

[62] Sharma S. S., Dietz K. J. (2009). The Relationship between Metal Toxicity and Cellular Redox Imbalance. *Trends in Plant Science*.

[63] Alhujaily M. (2024). Molecular Assessment of Methylglyoxal-Induced Toxicity and Therapeutic Approaches in Various Diseases: Exploring the Interplay with the Glyoxalase System. *The Life*.

[64] Hasanuzzaman M., Raihan M. R. H., Masud A. A. C. (2021). Regulation of Reactive Oxygen Species and Antioxidant Defense in Plants Under Salinity. *International Journal of Molecular Sciences*.

[65] Viana G. F. d. S., Garcia K. S., Menezes-Filho J. A. (2011). Assessment of Carcinogenic Heavy Metal Levels in Brazilian Cigarettes. *Environmental Monitoring and Assessment*.

[66] Arcury T. A., Trejo G., Moore D. (2020). It’s Worse to Breathe It Than to Smoke It: Secondhand Smoke Beliefs in a Group of Mexican and Central American Immigrants in the United States. *International Journal of Environmental Research and Public Health*.

[67] Ghoma W. E. O., Sevik H., Isinkaralar K. (2022). Using Indoor Plants as Biomonitors for Detection of Toxic Metals by Tobacco Smoke. *Air Quality, Atmosphere & Health*.

[68] Ugulu I. (2015). Determination of Heavy Metal Accumulation in Plant Samples by Spectrometric Techniques in Turkey. *Applied Spectroscopy Reviews*.

[69] Yang X., Guo S., Deng X., Xu D. (2021). Livelihood Adaptation of Rural Households Under Livelihood Stress: Evidence from Sichuan Province, China. *Agriculture*.

[70] Campos G. M., Vittinghoff E., Rabl C. (2009). Endoscopic and Surgical Treatments for Achalasia: A Systematic Review and Meta-Analysis. *Annals of Surgery*.

[71] Alves B. S. F., Pereira Junior J. B., Carvalho F. I. M., Dantas Filho H. A., Fernandes Dantas K. G. (2019). Mineral Composition of Amazonian Fruits by Flame Atomic Absorption Spectrometry Using Multivariate Analysis. *Biological Trace Element Research*.

[72] Chirigiu L., Popescu R., Bubulica M.-V., Popescu A. (2012). Determination of Chromium, Cooper, Iron, Zinc, Cadmium and Led by Graphite Furnace Atomic Absorption Spectrometry in Seven Phytopharmaceutical Products. *Revue Chimique*.

[73] Augustine A., Onwuka J., C Q. (2016). Determination of Heavy Metal Concentration in Neem (*Azadirachta indica*) Leaves, Bark and Soil along Some Major Roads in Lafia, Nasarawa State Nigeria. *Journal of Environmental Chemistry and Ecotoxicology*.

[74] Guo R., Qian R., Han F. (2023). Managing Straw and Nitrogen Fertilizer Based on Nitrate Threshold for Balancing Nitrogen Requirement of Maize and Nitrate Residue. *Journal of Environmental Management*.

[75] Liu W., Pan Q. S., Zhang P., Huang D. S., Fan A. P., Yi P. (2012). Determination of Total Arsenic in Chinese Traditional Herbs by High Pressure Digestion-Hydride Generation Atomic Fluorescence Spectrometry. *Advanced Materials Research*.

[76] Ozyigit I. I., Karahan F., Yalcin I. E., Hocaoglu-Ozyigit A., ilçim A. (2022). Heavy Metals and Trace Elements Detected in the Leaves of Medicinal Plants Collected in the Southeast Part of Turkey. *Arabian Journal of Geosciences*.

[77] Jurca T., Marian E., Vicas L., Braun M., Toth I. (2013). Analysis of Metal Content in Herbal Medicines. *Revue Chimique*.

[78] Guo C., Lv L., Liu Y. (2023). Applied Analytical Methods for Detecting Heavy Metals in Medicinal Plants. *Critical Reviews in Analytical Chemistry*.

[79] Wang Z., Li B., Hu Y. (2023). Characterization of Radix Angelicae Sinensis by Fluorescence and Near-Infrared Spectroscopies. *Analytical Letters*.

[80] Darko G., Dodd M., Nkansah M. A. (2017). Distribution and Ecological Risks of Toxic Metals in the Topsoils in the Kumasi Metropolis, Ghana. *Cogent Environmental Science*.

[81] Rashidimehr A., Mosavvari Z., Ziarati P., Eskandari S. (2023). Distribution, Accumulation, and Risk Assessment of Pb and Cd in the Tea Plant Leaves, Black Tea, and Soil from Different Tea Plantations in Lahijan, Iran. *Philippine Journal of Science*.

[82] Ali Taher M. (2003). Flame Atomic Absorption Spectrometric Determination of Trace Lead After Solid-Liquid Extraction and Preconcentration Using 1-(2-Pyridylazo)-2-Naphthol. *Croatica Chemica Acta*.

[83] Choudhury R. P., Acharya R., Nair A. G. C., Reddy A. V. R., Garg A. N. (2008). Availability of Essential Trace Elements in Medicinal Herbs Used for Diabetes Mellitus and Their Possible Correlations. *Journal of Radioanalytical and Nuclear Chemistry*.

[84] Bolann B. J., Rahil‐Khazen R., Henriksen H., Isrenn R., Ulvik R. J. (2007). Evaluation of Methods for Trace‐element Determination With Emphasis on Their Usability in the Clinical Routine Laboratory. *Scandinavian Journal of Clinical and Laboratory Investigation*.

[85] Gupta S., Pandotra P., Gupta A. P. (2010). Volatile (As and Hg) and Non-Volatile (Pb and Cd) Toxic Heavy Metals Analysis in Rhizome of *Zingiber officinale* Collected from Different Locations of North Western Himalayas by Atomic Absorption Spectroscopy. *Food and Chemical Toxicology*.

[86] J Potts P., T Ellis A., Kregsamer P., Streli C., West M., Wobrauschek P. (1999). X-ray Fluorescence Spectrometry. *Journal of Analytical Atomic Spectrometry*.

[87] Marguí E., Queralt I., de Almeida E. (2022). X-ray Fluorescence Spectrometry for Environmental Analysis: Basic Principles, Instrumentation, Applications and Recent Trends. *Chemosphere*.

[88] Chuparina E. V., Aisueva T. S. (2011). Determination of Heavy Metal Levels in Medicinal Plant Hemerocallis Minor Miller by x-ray Fluorescence Spectrometry. *Environmental Chemistry Letters*.

[89] Wilschefski S. C., Baxter M. R. (2019). Inductively Coupled Plasma Mass Spectrometry: Introduction to Analytical Aspects. *Clinical Biochemist Reviews*.

[90] Wojcieszek J., Kwiatkowski P., Ruzik L. (2017). Speciation Analysis and Bioaccessibility Evaluation of Trace Elements in Goji Berries (*Lycium barbarum*, L.). *Journal of Chromatography A*.

[91] Santos D., Nunes L. C., de Carvalho G. G. A. (2012). Laser-Induced Breakdown Spectroscopy for Analysis of Plant Materials: A Review. *Spectrochimica Acta Part B: Atomic Spectroscopy*.

[92] Saini S., Kaur N., Pati P. K. (2021). Phytohormones: Key Players in the Modulation of Heavy Metal Stress Tolerance in Plants. *Ecotoxicology and Environmental Safety*.

[93] Gautam M., Singh A. K. (2015). Impact of Climate Change on Water Resources. *Climate Change Modelling, Planning and Policy for Agriculture*.

[94] Morkunas I., Woźniak A., Mai V. C., Rucińska-Sobkowiak R., Jeandet P. (2018). The Role of Heavy Metals in Plant Response to Biotic Stress. *Molecules*.

[95] Roy M., McDonald L. M. (2015). Metal Uptake in Plants and Health Risk Assessments in Metal‐contaminated Smelter Soils. *Land Degradation & Development*.

[96] Berlowitz I., Torres E. G., Walt H., Wolf U., Maake C., Martin-Soelch C. (2020). Tobacco Is the Chief Medicinal Plant in My Work: Therapeutic Uses of Tobacco in Peruvian Amazonian Medicine Exemplified by the Work of a Maestro Tabaquero. *Frontiers in Pharmacology*.

[97] Ghori N. H., Ghori T., Hayat M. Q. (2019). Heavy Metal Stress and Responses in Plants. *International journal of Environmental Science and Technology*.

[98] Sharma G., Naushad M. (2020). Adsorptive Removal of Noxious Cadmium Ions from Aqueous Medium Using Activated Carbon/Zirconium Oxide Composite: Isotherm and Kinetic Modelling. *Journal of Molecular Liquids*.

[99] Naushad M., Alothman Z. A. (2015). Separation of Toxic Pb^2+^ Metal From Aqueous Solution Using Strongly Acidic Cation-Exchange Resin: Analytical Applications for the Removal of Metal Ions from Pharmaceutical Formulation. *Desalination and Water Treatment*.

[100] Liu L., Li W., Song W., Guo M. (2018). Remediation Techniques for Heavy Metal-Contaminated Soils: Principles and Applicability. *The Science of the Total Environment*.

[101] Sivaramakrishnan R., Incharoensakdi A. (2018). Utilization of Microalgae Feedstock for Concomitant Production of Bioethanol and Biodiesel. *Fuel*.

[102] Mathur P., Tripathi D. K., Baluška F., Mukherjee S. (2022). Auxin-Mediated Molecular Mechanisms of Heavy Metal and Metalloid Stress Regulation in Plants. *Environmental and Experimental Botany*.

[103] Tekere M. (2020). Biological Strategies for Heavy Metal Remediation. *Environmental Chemistry for a Sustainable World*.

[104] Narayanan M., Ali S. S., El-Sheekh M. (2023). A Comprehensive Review on the Potential of Microbial Enzymes in Multipollutant Bioremediation: Mechanisms, Challenges, and Future Prospects. *Journal of Environmental Management*.

[105] Mosa K. A., Saadoun I., Kumar K., Helmy M., Dhankher O. P. (2016). Potential Biotechnological Strategies for the Cleanup of Heavy Metals and Metalloids. *Frontiers of Plant Science*.

[106] Korver R. A., Koevoets I. T., Testerink C. (2018). Out of Shape during Stress: A Key Role for Auxin. *Trends in Plant Science*.

[107] Saini S., Sharma I., Pati P. K. (2017). Integrating the Knowledge of Auxin Homeostasis with Stress Tolerance in Plants. *Mechanism of Plant Hormone Signaling under Stress*.

[108] Ronzan M., Della Rovere F., Piacentini D., Fattorini L., Altamura M. M., Falasca G. Cadmium and Arsenic Affect Root Development in *Oryza sativa* L. Involving Auxin Jasmonate Crosstalk.

[109] Yuan H., Huang X. (2016). Inhibition of Root Meristem Growth by Cadmium Involves Nitric Oxide‐Mediated Repression of Auxin Accumulation and Signalling in Arabidopsis. *Plant, Cell and Environment*.

[110] Patra D. K., Pradhan C., Patra H. K. (2020). Toxic Metal Decontamination by Phytoremediation Approach: Concept, Challenges, Opportunities and Future Perspectives. *Environmental Technology & Innovation*.

[111] Shah V., Daverey A. (2020). Phytoremediation: A Multidisciplinary Approach to Clean up Heavy Metal Contaminated Soil. *Environmental Technology & Innovation*.

[112] Khalid S., Shahid M., Niazi N. K., Murtaza B., Bibi I., Dumat C. (2017). A Comparison of Technologies for Remediation of Heavy Metal Contaminated Soils. *Journal of Geochemical Exploration*.

[113] Ullah S., Liu Q., Wang S. (2023). Sources, Impacts, Factors Affecting Cr Uptake in Plants, and Mechanisms Behind Phytoremediation of Cr-Contaminated Soils. *Science of the Total Environment*.

[114] Leng L., Huang H., Li H., Li J., Zhou W. (2019). Biochar Stability Assessment Methods: A Review. *The Science of the Total Environment*.

[115] Wiedemeier D. B., Abiven S., Hockaday W. C. (2015). Aromaticity and Degree of Aromatic Condensation of Char. *Organic Geochemistry*.

[116] Zhang C., Wang W., Duan A. (2019). Adsorption Behavior of Engineered Carbons and Carbon Nanomaterials for Metal Endocrine Disruptors: Experiments and Theoretical Calculation. *Chemosphere*.

[117] Xu P., Sun C.-X., Ye X.-Z., Xiao W.-D., Zhang Q., Wang Q. (2016). The Effect of Biochar and Crop Straws on Heavy Metal Bioavailability and Plant Accumulation in a Cd and Pb Polluted Soil. *Ecotoxicology and Environmental Safety*.

[118] Nie C., Yang X., Niazi N. K. (2018). Impact of Sugarcane Bagasse-Derived Biochar on Heavy Metal Availability and Microbial Activity: A Field Study. *Chemosphere*.

[119] Khan M. A., Basir A., Fahad S. (2022). Biochar Optimizes Wheat Quality, Yield, and Nitrogen Acquisition in Low Fertile Calcareous Soil Treated With Organic and Mineral Nitrogen Fertilizers. *Frontiers in Plant Science*.

[120] Rajapaksha A. U., Vithanage M., Zhang M. (2014). Pyrolysis Condition Affected Sulfamethazine Sorption by Tea Waste Biochars. *Bioresource Technology*.

[121] Xu X., Cao X., Zhao L., Wang H., Yu H., Gao B. (2013). Removal of Cu, Zn, and Cd From Aqueous Solutions by the Dairy Manure-Derived Biochar. *Environmental Science & Pollution Research*.

[122] Kong H., He J., Gao Y., Wu H., Zhu X. (2011). Cosorption of Phenanthrene and Mercury(II) From Aqueous Solution by Soybean Stalk-Based Biochar. *Journal of Agricultural and Food Chemistry*.

[123] Samsuri A. W., Sadegh-Zadeh F., Seh-Bardan B. J. (2013). Adsorption of As(III) and As(V) by Fe Coated Biochars and Biochars Produced From Empty Fruit Bunch and Rice Husk. *Journal of Environmental Chemical Engineering*.

[124] Li H., Dong X., da Silva E. B., de Oliveira L. M., Chen Y., Ma L. Q. (2017). Mechanisms of Metal Sorption by Biochars: Biochar Characteristics and Modifications. *Chemosphere*.

[125] Wang Y. L., Liang Y. Z., Chen B. M. (2007). High-Performance Liquid Chromatography With Atmospheric Pressure Chemical Ionization and Electrospray Ionization Mass Spectrometry for Analysis of Angelica Sinensis. *Phytochemical Analysis*.

[126] Erdem H. (2021). The Effects of Biochars Produced in Different Pyrolsis Temperatures From Agricultural Wastes on Cadmium Uptake of Tobacco Plant. *Saudi Journal of Biological Sciences*.

[127] Mehmood I., Qiao L., Chen H., Tang Q., Woolf D., Fan M. (2020). Biochar Addition Leads to More Soil Organic Carbon Sequestration Under a Maize-Rice Cropping System Than Continuous Flooded Rice. *Agriculture, Ecosystems & Environment*.

[128] Sun C., Wang D., Shen X. (2020). Effects of Biochar, Compost and Straw Input on Root Exudation of Maize (*Zea mays* L.): From Function to Morphology. *Agriculture, Ecosystems & Environment*.

[129] Masud M. A. A., Shin W. S., Sarker A. (2023). A Critical Review of Sustainable Application of Biochar for Green Remediation: Research Uncertainty and Future Directions. *The Science of the Total Environment*.

[130] Cui X., Fang S., Yao Y. (2016). Potential Mechanisms of Cadmium Removal From Aqueous Solution by Canna Indica Derived Biochar. *The Science of the Total Environment*.

[131] Jagadeesh N., Sundaram B. (2023). Adsorption of Pollutants From Wastewater by Biochar: A Review. *Journal of Hazardous Materials Advances*.

[132] Burachevskaya M., Minkina T., Bauer T. (2023). Fabrication of Biochar Derived From Different Types of Feedstocks as an Efficient Adsorbent for Soil Heavy Metal Removal. *Scientific Reports*.

[133] Manikandan S. K., Pallavi P., Shetty K. (2023). Effective Usage of Biochar and Microorganisms for the Removal of Heavy Metal Ions and Pesticides. *Molecules*.

[134] Cho D. W., Chon C. M., Yim G. J. (2023). Adsorption of Potentially Harmful Elements by Metal-Biochar Prepared via Co-Pyrolysis of Coffee Grounds and Nano Fe (III) Oxides. *Chemosphere*.

[135] Gueret Yadiberet Menzembere E. R., He Y., Dong Y. (2023). Insight Into Modified Biochars and Their Immobilizing Effects on Heavy Metal (Loid) S in Contaminated Soils: Mechanisms and Influencing Factors. *Pedosphere*.

[136] Awang N. A., Wan Salleh W. N., Aziz F., Yusof N., Ismail A. F. (2023). A Review on Preparation, Surface Enhancement and Adsorption Mechanism of Biochar‐Supported Nano Zero‐Valent Iron Adsorbent for Hazardous Heavy Metals. *Journal of Chemical Technology and Biotechnology*.

[137] Muzyka R., Misztal E., Hrabak J., Banks S. W., Sajdak M. (2023). Various Biomass Pyrolysis Conditions Influence the Porosity and Pore Size Distribution of Biochar. *Energy*.

[138] Adhikari S., Moon E., Paz-Ferreiro J., Timms W. (2024). Comparative Analysis of Biochar Carbon Stability Methods and Implications for Carbon Credits. *Science of the Total Environment*.

[139] Zhang Z., Huang G., Zhang P., Shen J., Wang S., Li Y. (2023). Development of Iron-Based Biochar for Enhancing Nitrate Adsorption: Effects of Specific Surface Area, Electrostatic Force, and Functional Groups. *The Science of the Total Environment*.

[140] Arwenyo B., Varco J. J., Dygert A., Brown S., Pittman C. U., Mlsna T. (2023). Contribution of Modified P-Enriched Biochar on pH Buffering Capacity of Acidic Soil. *Journal of Environmental Management*.

[141] Balmuk G., Videgain M., Manyà J. J., Duman G., Yanik J. (2023). Effects of Pyrolysis Temperature and Pressure on Agronomic Properties of Biochar. *Journal of Analytical and Applied Pyrolysis*.

